# BaoShenTongLuo formula protects against podocyte injury by regulating AMPK-mediated mitochondrial biogenesis in diabetic kidney disease

**DOI:** 10.1186/s13020-023-00738-4

**Published:** 2023-03-26

**Authors:** Yifan Guo, Mengdi Wang, Yufei Liu, Yanyu Pang, Lei Tian, Jingwen Zhao, Mengchao Liu, Cun Shen, Yuan Meng, Yuefen Wang, Zhen Cai, Wenjing Zhao

**Affiliations:** 1 Department of Nephrology, Beijing Hospital of Traditional Chinese Medicine, Capital Medical University, Beijing, 100010 China; 2 Beijing University of Chinese Medicine, Beijing, 100029 China

**Keywords:** Diabetic kidney disease, AMPK, Mitochondrial biogenesis, Podocyte injury, BaoShenTongLuo formula

## Abstract

**Background:**

Mitochondrial dysfunction is considered to be an important contributor in podocyte injury under diabetic conditions. The BaoShenTongLuo (BSTL) formula has been shown to reduce podocyte damage and postpone the progression of diabetic kidney disease (DKD). The potential mechanisms underlying the effects of BSTL, however, have yet to be elucidated. In this study, we aimed to investigate whether the effects of BSTL are related to the regulation of mitochondrial biogenesis via the adenosine monophosphate-activated protein kinase (AMPK) pathway.

**Methods:**

High-Performance Liquid Chromatography Electrospray Ionization Mass Spectrometer (HPLC–ESI–MS) analysis was performed to investigate the characteristics of pure compounds in BSTL. db/db mice and mouse podocyte clone-5 (MPC5) cells were exposed to high glucose (HG) to induce DKD and podocyte damage. Body weight, random blood glucose, urinary albumin/creatinine ratio (UACR), indicators of renal function and renal histological lesions were measured. Markers of podocyte injury, mitochondrial morphology, mitochondrial deoxyribonucleic acid (mtDNA) content, mitochondrial respiratory chain complexes activities, reactive oxygen species (ROS) production, and mitochondrial membrane potential (MMP) levels were assessed. Protein expressions of AMPK, peroxisome proliferator-activated receptor gamma coactivator 1 alpha (PGC-1α), transcription factor A (TFAM), mitochondrial fusion protein 2 (MFN2) and dynamin-related protein 1 (DRP1) were also detected. MPC5 cells were transfected with AMPKα small interfering RNA (AMPKα siRNA) to determine the underlying mechanisms of BSTL improvement of mitochondrial function under diabetic conditions.

**Results:**

In vivo, treatment with BSTL reduced the UACR levels, reversed the histopathological changes in renal tissues, and alleviated the podocyte injury observed in db/db mice. After BSTL treatment, the decreased mtDNA content and mitochondrial respiratory chain complex I, III, and IV activities were significantly improved, and these effects were accompanied by maintenance of the protein expression of p-AMPKαT172, PGC-1α, TFAM and MFN2. The in vitro experiments also showed that BSTL reduced podocyte apoptosis, suppressed excessive cellular ROS production, and reversed the decreased in MMP that were observed under HG conditions. More importantly, the effects of BSTL in enhancing mitochondrial biogenesis and reducing podocyte apoptosis were inhibited in AMPKα siRNA-treated podocytes.

**Conclusion:**

BSTL plays a crucial role in protecting against podocyte injury by regulating the AMPK-mediated mitochondrial biogenesis in DKD.

## Introduction

DKD is the primary cause of end-stage renal disease (ESRD) worldwide [1]. Podocytes are essential in maintaining the glomerular filtration barrier's integrity and permselectivity [2]. Podocytes have limited capacities for proliferation and regeneration, and changes in these cells result in persistent and irreversible renal injury [3, 4]. Chronic hyperglycaemia triggers both morphological and functional abnormities in podocytes, which result in remodelling of the actin cytoskeleton, decreased protein expression of phenotype markers, hypertrophy, fusion or effacement of foot processes; these phenomena are followed by detachment, loss and apoptosis, thereby leading to proteinuria and subsequent damage [5]. Therefore, it is generally acknowledged that podocyte damage is crucial to the initiation and development of DKD.

Although multiple variables contribute to podocyte damage in DKD, the particular mechanisms are yet unknown. Recent studies have shown that mitochondria are essential for maintaining podocyte homeostasis [6]. Mitochondria, which are ‘power stations’ of cells, provide podocytes with adequate energy to maintain their specific structure and physiological functions [7], and abnormities in mitochondria results in podocyte injury [8]. Mitochondrial biogenesis, which is a complex cellular mechanism of self-protection that is initiated by pathological damage or insufficient energy supply, contributes to inner and outer mitochondrial membrane stability, new protein and lipid synthesis and mtDNA replication under the control of highly regulated transcriptional activities [9]. Recent studies have shown that the induction of mitochondrial biogenesis prevents oxidative damage and apoptosis in podocytes during DKD [10], indicating that mitochondrial biogenesis may play a protective role in podocytes. AMPK, which is a cellular energy sensor and regulator, is one of the most important molecules that positively regulates mitochondrial biogenesis [11]. Previous studies have shown that decreased AMPK activation disrupts mitochondrial biogenesis, eventually leading to impaired renal podocyte function and the development of albuminuria under diabetic conditions [12]. However, metformin and pioglitazone, which are AMPK agonists, ameliorate hyperglycaemia-induced ROS production and prevent renal damage by improving mitochondrial biogenesis [13]. Therefore, the AMPK pathway is a potential target for promoting mitochondrial biogenesis and repairing podocyte injury in DKD.

Increasing numbers of studies have shown that traditional Chinese medicines (TCMs) have been widely utilized to treat patients, and TCMs have been proved to be useful in the treatment of a number of renal diseases [14–19]. BSTL is a TCM prescription that is composed of *Astragalus membranaceus* (Huangqi), *Rehmannia glutinosa* (Dihuang), *Cuscuta chinensis* (Tusizi), *Artemisia Anomala* (Liujinu), *Euonymus alatus* (Guijianyu), *Hirudo* (Shuizhi) and *Salvia Miltiorrhiza* Bunge (Danshen). Previously, we demonstrated that BSTL significantly reduced 24 h urinary protein and serum creatinine (Scr) levels and improved renal function in patients with DKD [20, 21], and we further confirmed that BSTL inhibited podocyte apoptosis by regulating the phosphoinositide 3-kinase (PI3K)/protein kinase B (Akt) pathway in kk-Ay mice [22]. However, it remains unclear whether BSTL protects against podocyte damage by promoting mitochondrial biogenesis via the AMPK pathway under diabetic conditions. In the present study, we explored the role of BSTL in db/db mice and HG-treated podocytes and further elucidated the underlying molecular mechanisms.

## Materials and methods

### HPLC–ESI–MS analysis

SCIEX ExionLC AD system (SCIEX, Foster City, CA, USA) equipped with a solvent delivery system, a degasser, an autosampler, a column oven, and a controller was used for HPLC–ESI–MS analysis. After decocting BSTL formula pieces, the liquid was diluted 100 times, filtered, and formed the sample compounds. The separation of the compounds was performed on a Waters ACQUITY UPLC HSS T3 (2.1 × 100 mm, 1.8 μm) at 40 °C, using water (A) and acetonitrile (B) containing 0.05% formic acid as the mobile phase, with a flow-rate of 0.3 ml/min. The substances were ionized in the mass spectrometer's electrospray ionization (ESI) ion source and were detected in the selected ion recording (SIR) mode. The negative and positive ESI mode of mass spectra was acquired using the X500R Q-TOF system with a Twin Spray source (SCIEX, Foster City, CA, USA). The spectra for TOF–MS and TOF–MS/MS analysis covered the m/z ranges of 100-1,500 Da and 50-1,500 Da. SCIEX OS Software™ 2.0 (SCIEX, Foster City, CA, USA) was used to analyze the data.

### Animals and treatment

All animal experiments were carried out in accordance with the protocol authorized by the Ethics Committee of Beijing University of Chinese Medicine (BUCM-4-2020121804-4173). Male db/db mice and wild-type m/m mice (6 weeks of age) were purchased from Cavens Biogle Model Animal Research Co., Ltd. (certificate number: SCXK2016-0010). All mice were housed in Beijing University of Chinese Medicine's pathogen-free animal facility. The mice were housed in an environment with a 12/12 h light cycle, a humidity level of 60%, a temperature range of 22–24 °C, and unrestricted access to food and drink. After 2 weeks of adaptive feeding, the blood glucose levels of the mice were randomly measured, and two consecutive readings over 16.7 mmol/L were considered to indicate the successful establishment of the model; db/db mice that met this criterion were used for further research. Then, db/db mice were randomly divided into the model group (db/db, n = 8) or BSTL group (db/db + B, n = 8) using the random number approach, and the m/m mice were placed in the nondiabetic control group (con, n = 8). The mice in the BSTL group were given BSTL by intragastric administration, and the BSTL extract was provided by the pharmacy of Beijing Hospital of Traditional Chinese Medicine, Capital Medical University, as reported in our previous study [22]. The dosage of the crude BSTL drug that was administered to the mice was 16.5 g/kg/d; this dosage represented the regular dosage given to human adults. The mice in the control and model groups were given the same volume of distilled water. Body weight and random blood glucose levels in tail blood were tested every 2 weeks. In the case of water supplied only, the 8 h urine volume of mice was collected to evaluate the urinary albumin level every 4 weeks. The mice were killed after 12 weeks of treatment. Serum was collected, and kidneys were harvested for further analysis.

### Preparation of drug-containing serum

Forty male Sprague-Dawley (SD) rats (8 weeks of age and weighing 200 ± 30 g) were purchased from Beijing Huafukang Biotechnology Co., Ltd., and after one week of adaptive feeding, all the SD rats were randomly assigned to either the BSTL or blank groups, with 20 rats in each group. The rats in the BSTL group were given BSTL at a dosage of 36.6 g/kg/days and the rats in the blank group were given an equal volume of distilled water for 7 consecutive days. Then, the rats were anaesthetized with 1% pentobarbital sodium at a dose of 40 mg/kg, and 5–8 ml of blood was collected from the abdominal aorta of each rat. Serum samples from the same group were pooled after being centrifuged at 3,000 rpm for 10 min. After incubation 30 min in a 56 °C water bath, the serum was filtered, rebottled into 1.5 ml sterile centrifuge tubes, and stored at − 20 °C.

### Cell culture and treatment

MPC5 cells were donated by Prof. Weijing Liu (Dongzhimen Hospital, Beijing University of Traditional Chinese Medicine, China). Podocytes were cultured at 33 °C in medium that consisted of Roswell Park Memorial Institute (RPMI) 1640 medium (11879020/11875093, Gibco, NY, USA) supplemented with 10% foetal bovine serum (10099-141, Gibco), 100 μg/mL streptomycin, 100 U/mL penicillin G (V900929, Sigma, MO, USA), and 100 U/mL recombinant murine interferon (IFN)-γ (315-05-20, PeproTech, NJ, USA) to facilitate proliferation. Then, the podocytes were cultured at 37 °C for 10–14 days in RPMI 1640 medium without IFN-γ to facilitate cell differentiation. The podocytes were used for the in vitro experiment when the confluence was approximately 80%. The differentiated cells were stimulated for 48 h with normal glucose (NG, 5.5 mM), HG (HG, 30 mM), and BSTL (H + B, 30 mM glucose + BSTL drug-containing serum). All experimental results were verified in at least three independent podocyte cultures.

### Biochemical indicator measurements

A mouse albumin ELISA kit (ab108792, Abcam, OR, USA) and creatinine assay kit (C011-2-1) were used to measure the urinary albumin and urine creatinine levels, and then, the results were used to calculate the UACR. Scr, blood urea nitrogen (BUN), alanine aminotransferase (ALT), and aspartate aminotransferase (AST) levels were measured with the creatinine assay kit (C011-2-1), urea assay kit (C013-1-1), ALT assay kit (C009-2-1) and AST assay kit (C010-2-1) from Nanjing Jiancheng Biotechnology Co., Ltd. (JiangSu, China) according to the manufacturer’s instructions.

### Renal histological examination

Kidney samples were fixed with 4% paraformaldehyde and incubated at 4 °C for 72 h. The process of dehydration was carried out by a completely automated closed tissue dewatering machine according to standard procedures. Then, the samples were embedded in paraffin and cut into 2–3-μm-thick sections. Haematoxylin and eosin (HE), periodic acid-Schiff (PAS), and Masson staining were performed by the Department of Pathology, Beijing Hospital of Traditional Chinese Medicine, Capital Medical University. The slides were scanned and viewed with a Leica (Aperio CS2, Germany). At least ten randomly chosen fields for each mouse were evaluated under the microscope and analysed with Image-Pro Plus 6.0 software.

### Immunohistochemical (IHC) staining

Paraffin-embedded kidney sections were incubated at 60 °C for 60 min, deparaffined with xylene three times for 15 min each, hydrated with gradient ethanol solution for 5 min each, and finally immersed in deionized water. Following a 20 min incubation at 95 °C with an antigen retrieval solution for antigen retrieval, the sections were let to cool naturally to room temperature. The endogenous peroxidase activity was quenched by incubation with 3% H_2_O_2_ at room temperature for 10 min. The sections were then blocked with goat serum (ZLI-9056, ZSBIO company, Beijing, China) at 37 °C for 30 min, followed by incubation with anti-nephrin (1:2000, ab216341, Abcam), anti-podocin (1:1000, ab50339, Abcam) and anti-cleaved caspase-3 (1:400, 19677-1-AP, Proteintech, Wuhan, China) antibodies overnight at 4 °C. The sections were then washed in phosphate-buffered saline (PBS), treated for 20 min at room temperature with horseradish peroxidase-conjugated anti-rabbit secondary antibody (PV-9001, ZSBIO business), and colour development was performed by incubating with diaminobenzidine (DAB, ZLI-9018, ZSBIO company). Finally, the nuclei were stained with haematoxylin. At least five randomly chosen fields for each sample were evaluated under the microscope and analysed with Image-Pro Plus 6.0 software.

### Transmission electron microscopy (TEM)

The mouse kidneys were cut into rectangular strips with a volume of approximately 1 mm^3^, fixed in 2.5% glutaraldehyde solution at 4 °C for 4 h, and washed three times with 0.1 mol/L phosphate buffer for 15 min each. Then the samples were fixed with 1% citrate solution for 2 h, washed three times with 0.1 mol/L phosphate buffer again, and dehydrated in a series of ethanol solutions (15 min each in 30%, 50%, 70%, 90%, 95% and 100% ethanol) followed by 100% acetone before hardening with epoxy resin (Epon) for 9 h. The samples were cut into 50–70-nm-thick ultrathin sections by a Reichert-Jung Ultracut S Ultramicrotome (Leica EM UC7, Germany). Then, the sections were stained with 2% uranyl acetate and lead citrate and observed with high-resolution TEM (JEM-1400 Plus, Japan).

### Immunofluorescence assay

Paraffin-embedded kidney sections were deparaffinized and hydrated, and antigens were retrieved as described for IHC staining. Then, the sections were permeabilized by incubation with 0.3% phosphate-buffered solution (PBST) for 10 min, followed by incubation with 3% donkey serum for 30 min at 37 °C. The sections were then incubated with a mixture of rabbit anti-phospho-adenosine monophosphate-activated protein kinase alpha (p-AMPKα, 1:100, AF3423, Affinity, NJ, USA) antibody and mouse anti-synaptopodin (1:200, sc515842, Santa Cruz Biotechnology, CA, USA) antibody overnight at 4 °C. After washing with PBS, the sections were stained with a mixture of Alexa Fluor 488-conjugated donkey anti-mouse IgG (1:2000, A21206, Invitrogen, PA, USA) and Alexa Fluor 594-conjugated donkey anti-rabbit IgG (1:2000, A32754, Invitrogen) as the secondary antibodies at 37 °C for 60 min. The nuclei were counterstained with 4’,6-diamidino-2-phenylindole (DAPI, ZL1-9557, ZSBIO company). In vitro, podocytes were cultured in 24-well plates and treated with different media (NG, HG, HG + B) for 48 h. After fixation with 4% paraformaldehyde, the cells were permeabilized with 0.3% Triton X-100 and blocked with 3% donkey serum. Then, the cells were stained with a rabbit anti-p-AMPKα antibody or mouse anti-translocase of outer mitochondrial membrane 20 homologue antibody (TOM20, 1:200, sc17764, Santa Cruz Biotechnology) overnight at 4 °C. The podocyte slides were stained with a mixture of Alexa Fluor 488-conjguated donkey anti-mouse IgG (1:2000) or Alexa Fluor 594-conjguated donkey anti-rabbit IgG (1:2000) as secondary antibodies at 37 °C for 60 min, and then, the nuclei were counterstained with DAPI. A fluorescence microscope (A1 HAL 100, ZEISS Scope, Germany) was used to observe the slides and capture microscopic images, and Image-Pro Plus 6.0 software was used to analyse and quantify the data.

### Phalloidin staining

Podocytes were cultured, treated, fixed, permeabilized, and blocked as described for the immunofluorescence assay. Then, podocytes were incubated with phalloidin (1:5000, P5282, Sigma) in the dark for 40 min at 37 °C, and the nuclei were counterstained with DAPI. The cells were observed and microscopic images were recorded by fluorescence microscopy (A1 HAL 100, ZEISS Scope, Germany).

### MitoTracker staining

Podocytes were cultured and treated as described for the immunofluorescence assay. Subsequently, the cells were stained with MitoTracker red (1:2000, 8778P, CST, MA, USA) following the manufacturer’s instructions. Fluorescence images were captured with a confocal microscope (DCM-3D, Leica, Germany).

### Terminal Deoxynucleotidyl Transferase-Mediated dUTP-biotin Nick end Labelling (TUNEL) analysis

The DeadEnd™ Colorimetric TUNEL System (G7130/G7160, Promega, WI, USA) was used to identify apoptotic glomerular cells in vivo. Paraffin-embedded kidney sections were deparaffinized and hydrated as described for IHC staining. Then, the sections were treated with proteinase K for 15 min, washed with PBS, and fixed with 4% paraformaldehyde, followed by equilibration for 10 min at room temperature. The sections were incubated with r-terminal deoxynucleotidyl transferase (rTdT) reaction mixture for 1 h at 37 °C and immersed in 2X saline sodium citrate (SSC) for 15 min at room temperature. After washing with PBS, the sections were incubated with 3% hydrogen peroxide for 5 min at room temperature, incubated with streptavidin horseradish peroxidase (HRP) solution and visualized with DAB. Finally, the sections were blocked with 100% glycerin. An in vitro in situ cell death detection kit (11684817910, Roche, BASEL, SWZ) was used to assess podocyte apoptosis following the manufacturer’s protocol. Podocytes were cultured in 6-well plates and treated with different media for 48 h. Then, they were incubated with the TUNEL reaction mixture for 1 h at 37 °C, washed with PBS, and counterstained with DAPI. The apoptotic cells in the kidney sections were observed with a Leica microscope (Aperio CS2, Germany), and the apoptotic podocytes were observed under a fluorescence microscope (CKX41, OLYMPUS). Image-Pro Plus 6.0 software was used to analyse and quantify the data.

### Mitochondrial respiratory chain complex measurement

Kidney tissues were minced, and the activities of mitochondrial respiratory chain complexes I, III, and IV were measured using mitochondrial respiratory chain complex I, III, and IV activity detection kits (BC0515/BC3245/BC0945, Solarbio, Beijing, China) according to the manufacturer’s instructions. Podocytes were cultured in 6-well plates and treated with different media for 48 h. After digestion with trypsin, the podocytes were collected and used for analysis following the instructions.

### Flow cytometry

Podocytes were cultured in 6-well plates and treated with different media for 48 h. The level of podocyte apoptosis was measured via an Annexin V-fluorescein isothiocyanate (FITC) apoptosis detection kit (556547, Becton Dickinson and Company, NY, USA). Briefly, the density of podocytes was adjusted to 1 × 10^6^ cells/mL, and then, 5 μl Annexin V-FITC and 5 μl propidium iodide (PI) were added to 100 μl cell suspensions. After incubation at room temperature for 30 min in the dark, podocytes were centrifuged and resuspended in binding buffer. The MMP level was measured via a JC-1 assay kit (C2006, Beyotime Biotechnology, Shanghai, China). Briefly, 100 μl of cell suspensions were collected and incubated with JC-1 working solution at 37 °C in the dark for 20 min. Then, the podocytes were washed with JC-1 staining buffer (1X) and resuspended in binding buffer. The generation of ROS by podocytes was measured using the 2’,7’-dichlorodihydrofluorescein diacetate (DCFH-DA) activity assay kit (red) (MAK145, Sigma), which includes an intracellular ROS fluorescent probe. Briefly, the cell suspension was collected and centrifuged, and then podocytes were resuspended in ROS working solution and incubated at room temperature in the dark for 20 min. A flow cytometer (Calibur II, Becton, Dickinson and Company, USA) was used to analyse the podocytes from the different groups, and FlowJo software was used to analyse the levels of Annexin V-FITC/PI, MMP and ROS.

### AMPKα siRNA transfection

AMPKα siRNA (45313, Santa Cruz Biotechnology) was transfected into cells with the Lipofectamine® RNAiMAX transfection kit (13778150, Invitrogen) according to the manufacturer's protocol. Briefly, podocytes were cultured in 6-well plates for 24 h. RNAiMAX transfection reagent and AMPKα siRNA were added to the reaction mixture. Then, the podocytes were cultured with serum-free medium and reaction mixture for 24 h, and the medium was changed for 6–8 h. After the transfection was completed, the podocytes were treated for 48 h with various mediums. The protein level of AMPKα was measured by western blotting analysis to confirm transfection success. Podocytes were collected 24 h after transfection for the following experiments.

### Western blotting (WB) analysis

The renal cortex tissues and podocytes were lysed with radio immunoprecipitation assay lysis buffer (1:50, C1053, Applygen, Beijing, China) supplemented with protease inhibitors and protein phosphorylase inhibitors (1:100, P1260, Applygen). The renal cortex tissues were cut into pieces, and podocytes were harvested by scraping with a cell scraper; then, the samples were lysed via intermittent ultrasound for 3 min. After centrifugation at 15000 rpm at 4 °C for 15 min, the supernatants were harvested to measure the protein concentration at 562 nm with the bicinchoninic acid (BCA) protein quantification kit (P1511, Applygen). The protein extraction solution was diluted with loading buffer (5X), and the samples were incubated at 95 °C for 15 min, aliquoted, and stored at − 80 °C. WB analysis was performed using a standard protocol. Briefly, markers and samples were added to the designated wells of electrophoresis gels. The proteins were transferred to membranes after electrophoresis and then blocked. The anti-nephrin (1:1000, ab216341, Abcam), anti-podocin (1:1500, ab50339, Abcam), anti-cleaved caspase-3 (1:1000, 19677-1-AP, Proteintech), anti-PGC-1α (1:500, AF5395, Affinity), anti-TFAM (1:2000, ab131607, Abcam), anti-DRP1 (1:1000, ab184247, Abcam), anti-MFN2 (1:1000, 9482S, CST), anti-AMPKα (1:1000, 5831 T, CST), anti-p-AMPKα (1:1000, 2535S, CST) and glyceraldehyde phosphate dehydrogenase (GAPDH, 1:5000, 10494-1-AP, Proteintech) primary antibodies were added to the membranes and incubated at 4 °C overnight. After washing, the membranes were incubated with goat anti-rabbit IgG (1:5000, C1309, Applygen) secondary antibody at room temperature for 1 h, and excess secondary antibodies were removed with western washing buffer. The bands were visualized by enhanced chemiluminescence (ECL) hypersensitive luminescence solution in a dark room, and the densitometry values were measured with ImageJ.

### Quantification of mitochondrial DNA

The relative copy number of mtDNA was determined based on the ratio of mtDNA to nuclear DNA (nDNA) and measured by qPCR assay. Cytochrome b (Cyt B) and cytochrome c oxidase subunit II (COII) were used as controls for mtDNA, and GAPDH was used as a control for nDNA. The primer sequences (synthesized by Bao Biological Engineering Co., LTD, Dalian, China) are listed in Table 1. Total DNA was extracted from renal tissues and podocytes with a universal genomic DNA purification mini spin kit (D0063, Beyotime Biotechnology) according to the manufacturer's instructions. A Talent quantitative real-time PCR (qPCR) kit (RR003Q, Bao Biological Engineering Co., Ltd., Dalian, China) was used to perform the qPCRs (5 min denaturation step at 95 °C, then 40 cycles of 10 s at 95 °C, 30 s at 60 °C and 30 s at 70 °C) using a Fast Real-Time PCR system (Roche, Switzerland). The 2^−ΔΔCt^ method was used to calculate the relative expression.

**Table 1:** Primer information

Gene symbol	Primers	Sequence (5′ → 3′)	Gene ID	Product Length (bps)
COII	Forward	ACCTGGTGAACTACGACTGCTAGA	NC_005089.1	184 bp
	Reverse	CCCTGGTCGGTTTGATGTTACTGT		
Cyt B	Forward	TTCGCAGTCATAGCCACAGCATT	NC_005089.1	242 bp
	Reverse	TGGAGGAAGAGGAGGTGAACGATT		
GAPDH	Forward	GAAGGTGGTGAAGCAGGCATCT	NC_000072.7	116 bp
	Reverse	CGGCATCGAAGGTGGAAGAGTG		

### Statistical analysis

Normally distributed data were presented as mean ± standard deviation. One-way analysis of variance (ANOVA) was used for multiple group comparisons, and the least significant difference (LSD) test was used for pairwise comparisons. While skewed distribution data were presented as the median and interquartile range (IQR) and compared using the nonparametric test. Wilcoxon Rank Sum test was used to compare the differences between the two groups. The data came from at least three separate tests. *P* < *0.05* was considered statistically significant. Statistical and data analyses were performed with International Business Machines Corporation Statistical Product and Service solutions (IBM SPSS) 26.0 software. The graphical results were analysed using GraphPad Prism 7.0, and composite figures were generated with Adobe Illustrator CC 2018.

## Results

### Characteristics of pure compounds in BSTL

**Fig. 1:**
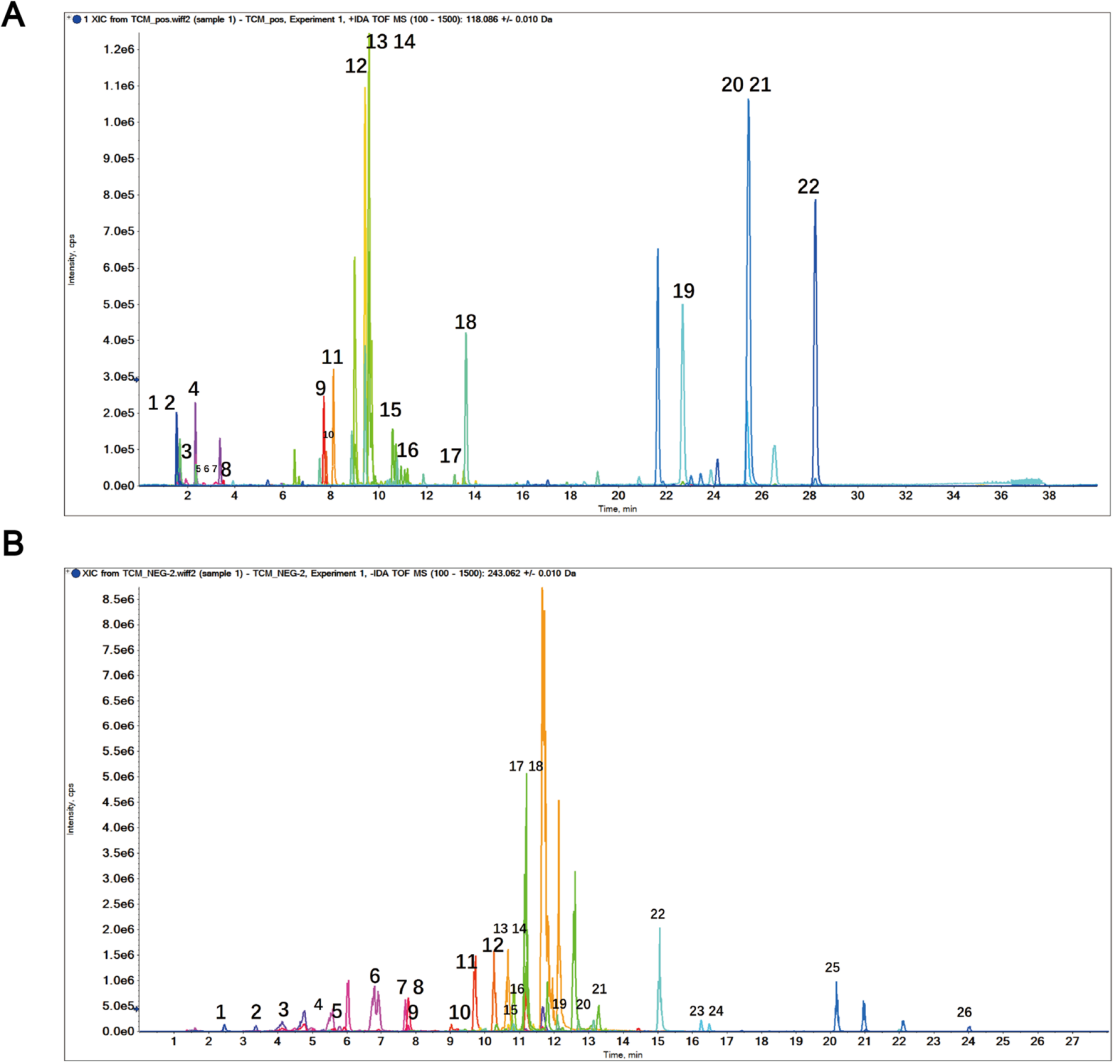
On chromatograms of BSTL analyzed by HPLC–ESI–MS analysis. **A** Positive base peak mass spectrometry spectrum of BSTL. **B** Negative base peak mass spectrometry spectrum of BSTL

HPLC–ESI–MS analysis is a modern, rapid, and sensitive method for drug analysis, which has been widely used in the analysis and identification of TCM components. In our study, BSTL analysis was conducted in both positive and negative ESI modes. As shown in Fig. 1, the positive base peak and negative base peak were displayed in the MS spectrum. There were 48 kinds of substances of BSTL identified by MS. The identified compounds were displayed in Tables 2 and 3, including salvianolic acid, tanshinones, astragalosides, and rehmanniosides. More importantly, many substances such as cryptotanshinone (number 21, Fig. 1A), tanshinone IIA (number 22, Fig. 1A), rosmarinic acid (number 17, Fig. 1B), salvianolic acid A (number 18, Fig. 1B) and apigenin (number 22, Fig. 1B) might present the biological activity of improving mitochondrial dysfunction [23–27]. Therefore, our research furture studied the role and mechanism of BSTL in mitochondrial biogenesis.

**Table 2:** Positive base peak chemical components of BSTL identified by HPLC-ESI/MS

No	Retention time	Formula	Experimental mass	Identification
1	1.54	C5H11NO2	118.0859	Betaine
2	1.56	C7H7NO2	138.055	Trigonelline
3	1.64	C7H13NO2	144.1019	Stachydrine
4	2.33	C5H5N5	136.0615	Adenine
5	2.35	C5H4N4O	137.0458	6-Hydroxypurine
6	2.35	C6H5NO2	124.0390	Nicotinic acid
7	2.70	C6H13NO2	132.1018	Isoleucine
8	3.52	C10H13N5O3	252.1089	Cordycepin
9	7.71	C16H24NO5	310.1645	Sinapine
10	7.80	C17H20N4O6	377.1455	Vitamin B2
11	8.11	C20H24NO4	342.1697	Magnoflorine
12	9.43	C22H22O10	447.1281	Calycosin-7-O-glucoside
13	9.59	C15H10O7	303.0497	Quercetin
14	9.59	C21H20O12	465.1025	Hyperin
15	10.58	C16H12O7	317.0655	Isorhamnetin
16	10.92	C22H22O11	463.1239	Pratensein-7-O-glucoside
17	13.15	C17H18O5	303.1227	Isomucronulatol
18	13.64	C16H12O5	285.0755	Calycosin
19	22.69	C18H14O3	279.1013	Dihydrotanshinone I
20	25.37	C18H12O3	277.0858	Tanshinone I
21	25.44	C19H20O3	297.1483	Cryptotanshinone
22	28.22	C19H18O3	295.1326	Tanshinone IIA

**Table 3:** Negative base peak chemical components of BSTL identified by HPLC-ESI/MS

No	Retention time	Formula	Experimental mass	Identification
1	2.45	C9H12N2O6	243.0620	Uridine
2	3.36	C27H42O20	685.2193	Rehmannioside D
3	4.12	C9H10O5	197.0455	Danshensu
4	5.55	C16H18O9	353.0875	Neochlorogenic acid
5	5.78	C11H12N2O2	203.0824	L-Tryptophan
6	6.79	C16H18O9	353.0873	Chlorogenic acid
7	7.70	C17H24O10.HCOOH	433.1348	Geniposide
8	7.78	C22H30O14	517.1561	Sibiricose A5
9	7.79	C9H8O4	179.0347	Caffeic acid
10	9.17	C22H18O12	473.0728	Cichoric acid
11	9.71	C21H18O12	461.0724	Luteolin-7-O-Glucuronide
12	10.26	C20H18O10	417.0824	Salvianolic acid D
13	10.67	C36H30O16	717.1455	Salvianolic acid B
14	10.68	C30H38O15	637.2138	Leucosceptoside A
15	10.79	C21H20O10	431.0988	Genistin
16	10.83	C21H18O11	445.0773	Baicalin
17	11.18	C18H16O8	359.0771	Rosmarinic acid
18	11.81	C26H22O10	493.1138	Salvianolic acid A
19	12.10	C31H40O15	651.2297	Rehmannioside
20	12.71	C31H40O15	651.2298	Epimeredinoside A
21	13.14	C23H28O10	463.1612	Isomucronulatol-7-O-glucoside
22	15.05	C15H10O5	269.0453	Apigenin
23	16.25	C41H68O14.HCOOH	829.4587	Astragaloside IV
24	16.61	C42H66O14	793.4386	Chikusetsusponin IVa
25	20.19	C45H72O16.HCOOH	913.4797	Astragaloside I
26	24.02	C48H76O19	955.4906	Ginsenoside-Ro

### BSTL reduced proteinuria and renal histological damage in db/db mice

**Fig. 2:**
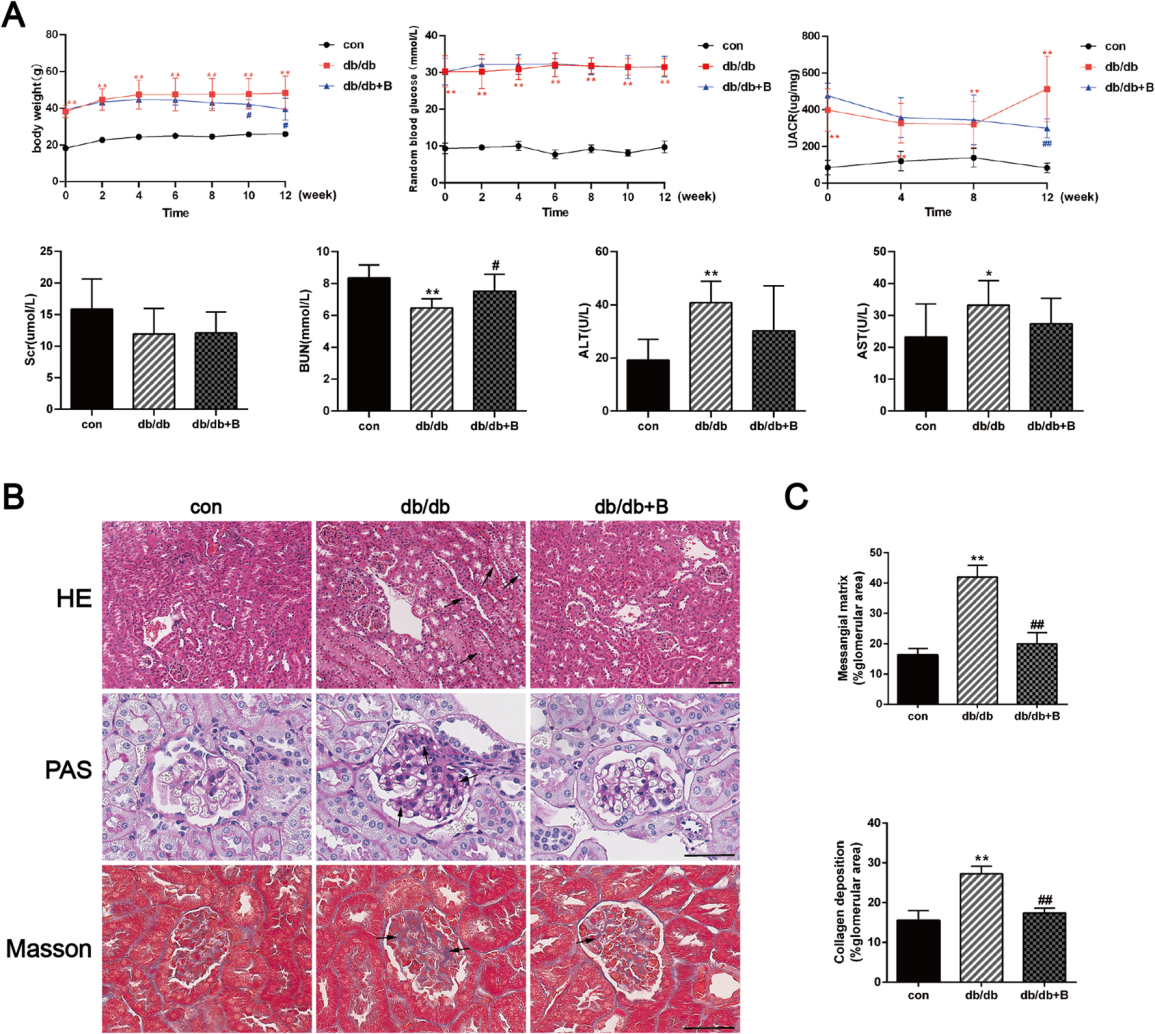
Effect of BSTL on biochemical indicators and renal histological changes in db/db mice. **A** Quantitative assessment of body weight, random blood glucose, UACR, Scr, BUN, ALT and AST of mice. **B** Representative micrographs of HE-stained kidney sections (× 200), PAS-stained kidney sections (× 400), and Masson’s trichrome-stained kidney sections (× 400) from different groups. Scale bar, 30 µm. The arrows indicate representative pathological changes. **C** Quantitative assessment of mesangial matrix by PAS staining and collagen deposition in glomeruli. ^*^P < 0.05 vs. con; ^**^P < 0.01 vs. con; ^#^*P* < 0.05 vs. db/db; ^##^*P* < 0.01 vs. db/db. con, control mice; db/db, db/db mice; db/db + B, db/db mice treated with BSTL

As shown in Fig. 2A, db/db mice had higher body weight, random blood glucose levels, and UACR as well as lower BUN levels than the control mice; however, the Scr levels were not significantly different. BSTL treatment caused a significant decrease in the UACR, a mild decrease in body weight, and only a slight recovery of BUN levels, while there were no significant changes in blood glucose and Scr levels, compared with those in the untreated db/db mice at week 12. The levels of ALT and AST were significantly increased in db/db mice, indicating metabolism-related liver damage. However, BSTL slightly restored the levels of ALT and AST in db/db mice. Histological analysis revealed glomerular hypertrophy, thickening of the glomerular basement membrane, mesangial expansion with increased matrix and collagen deposition in glomeruli, particle and vacuole denaturation and lumen dilation in the tubules of db/db mice, and BSTL treatment significantly improved these pathological changes (Fig. 2B, C).

### BSTL protected against podocyte injury in db/db mice and HG-treated podocytes

**Fig. 3:**
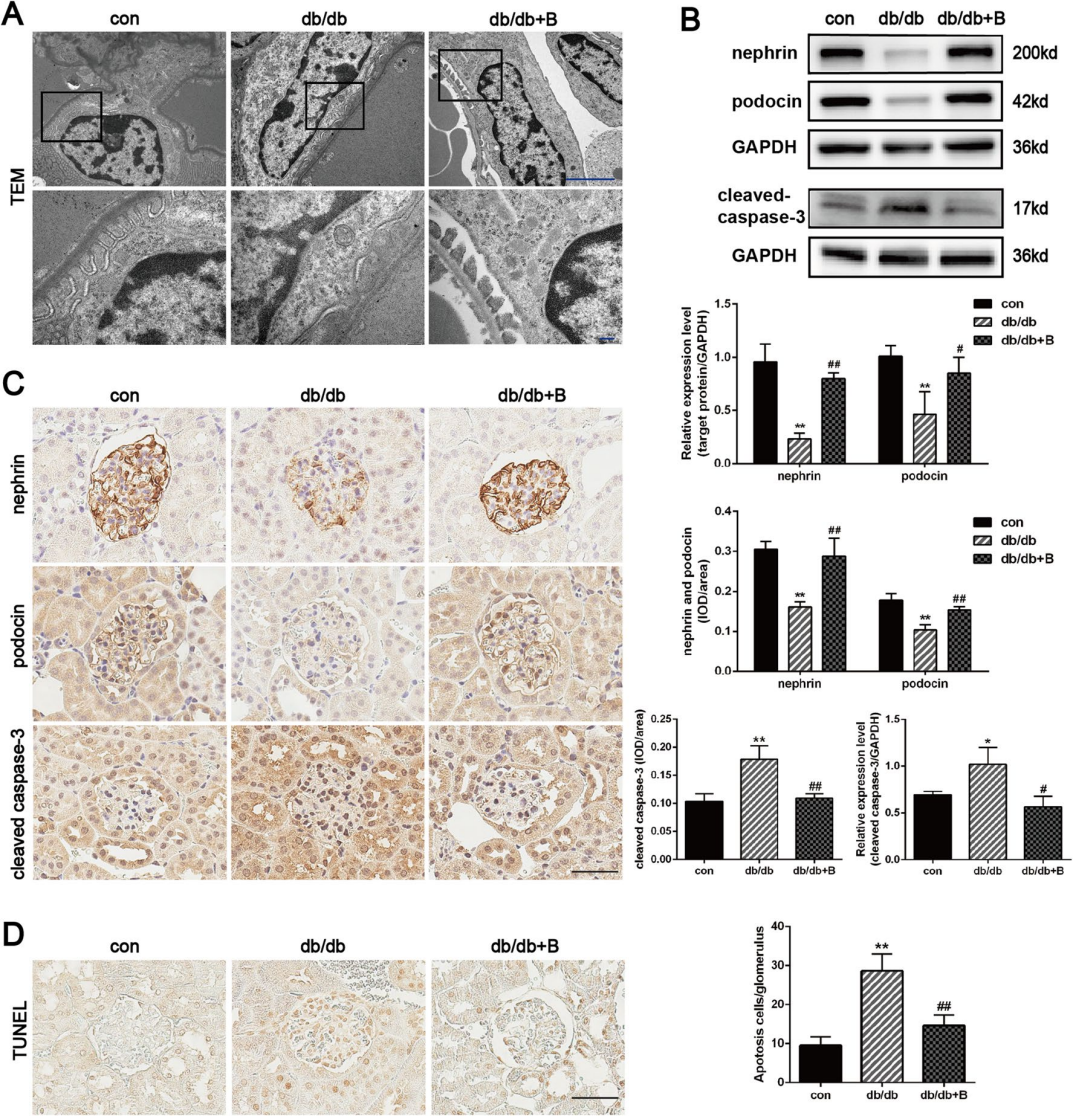
Effect of BSTL on podocyte injury in vivo. **A** Representative TEM micrographs of the kidneys of mice from different groups (× 15 000, × 50 000). Scale bar, 1 µm. **B** Representative western blots and quantitative assessment of nephrin, podocin, and cleaved caspase-3 in the kidneys of mice. **C** IHC staining of renal sections and quantitative assessment for nephrin, podocin, and cleaved caspase-3 (× 400). Scale bar, 30 µm. **D** TUNEL staining and quantitative assessment in renal sections from different groups (× 400). Scale bar, 30 µm. ^*^P < 0.05 vs. con; ^**^P < 0.01 vs. con; ^#^*P* < 0.05 vs. db/db; ^##^*P* < 0.01 vs. db/db. con, control mice; db/db, db/db mice; db/db + B, db/db mice treated with BSTL

**Fig. 4:**
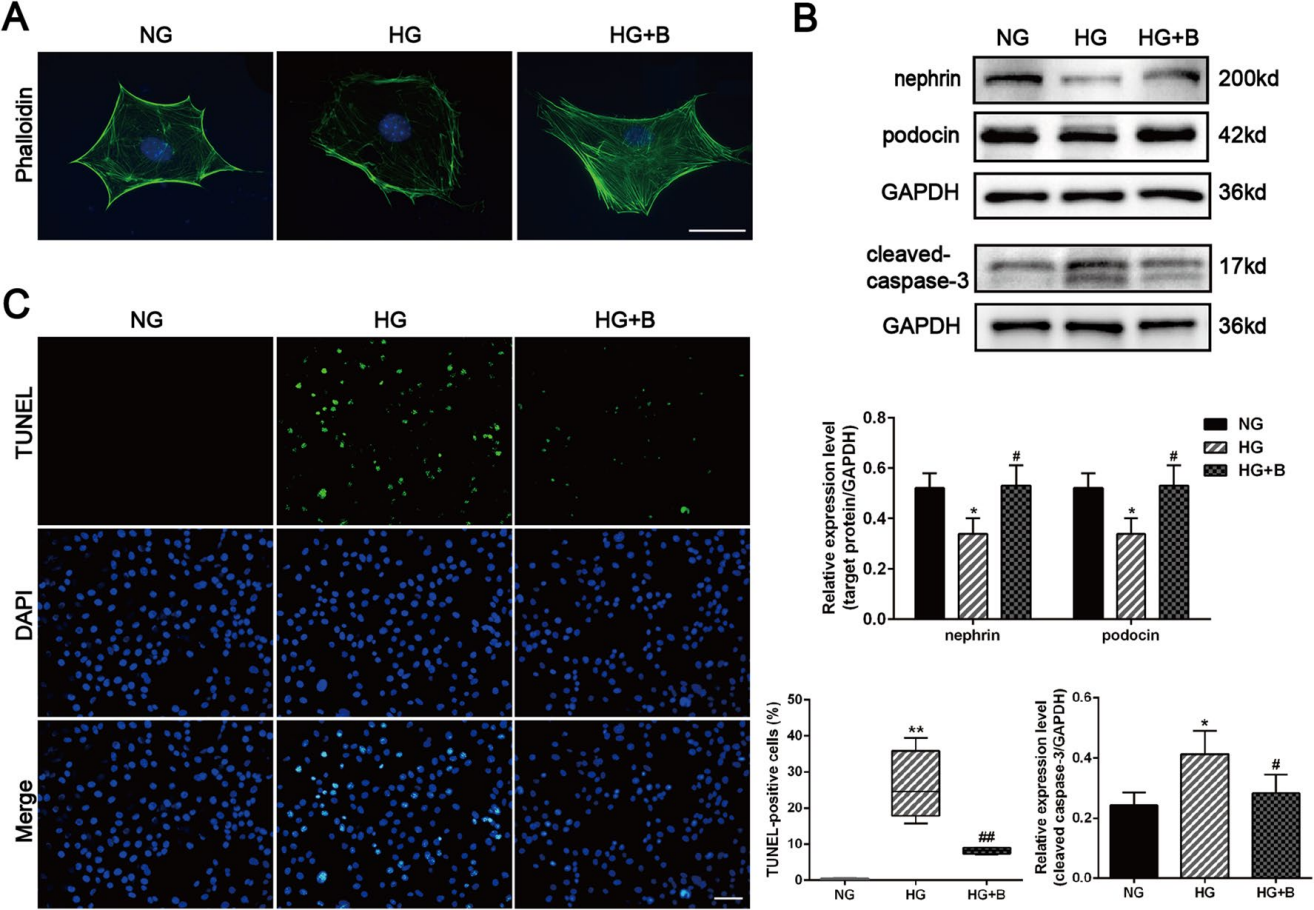
Effect of BSTL on podocyte injury in vitro. **A** Representative micrographs of phalloidin stained cytoskeletal microfilaments of podocytes from different groups (× 400). Scale bar, 30 µm. **B** Representative western blots and quantitative assessment of nephrin, podocin, and cleaved caspase-3 in podocytes from different groups. **C** TUNEL staining and quantitative assessment in podocytes from different groups (× 200). Scale bar, 30 µm. ^*^P < 0.05 vs. NG; ^**^P < 0.01 vs. NG; ^#^*P* < 0.05 vs. HG; ^##^*P* < 0.01 vs. HG. NG, normal glucose; HG, high glucose; HG + B, high glucose combined with BSTL drug-containing serum

TEM examination revealed podocyte injury in the glomeruli of db/db mice, which included podocyte foot process broadening and effacement; these phenomena were alleviated by BSTL treatment (Fig. 3A). According to phalloidin staining, HG-treated podocytes exhibited polygonal cellular shapes, which were related to a decrease in actin stress fibre content and accompanied by the formation of cortical F-actin rings in the cytoplasm. However, these structural changes in podocytes were reversed by BSTL treatment (Fig. 4A).

In parallel with the changes in podocyte structure, the expression of the protein components of filtration slits, namely, nephrin and podocin, was significantly reduced in diabetic kidneys, and this effect was reversed by BSTL treatment (Fig. 3B, C). The TUNEL assay showed that the number of apoptotic cells in the glomeruli was significantly increased in db/db mice compared with the control mice; WB and IHC staining revealed higher protein levels of cleaved caspase-3 in the kidneys of db/db mice than those of the control mice. BSTL markedly reduced the apoptotic cell numbers and cleaved caspase-3 expression (Fig. 3B–D). Similar to that in the kidneys of db/db mice, the expression of nephrin and podocin was also reduced in HG-treated podocytes. Consistently, HG significantly exacerbated apoptosis and increased the protein level of cleaved caspase-3 in podocytes, and these effects were nearly completely blocked by BSTL (Fig. 4B, C).

### BSTL improved mitochondrial biogenesis and dysfunction in db/db mice and HG-treated podocytes

**Fig. 5:**
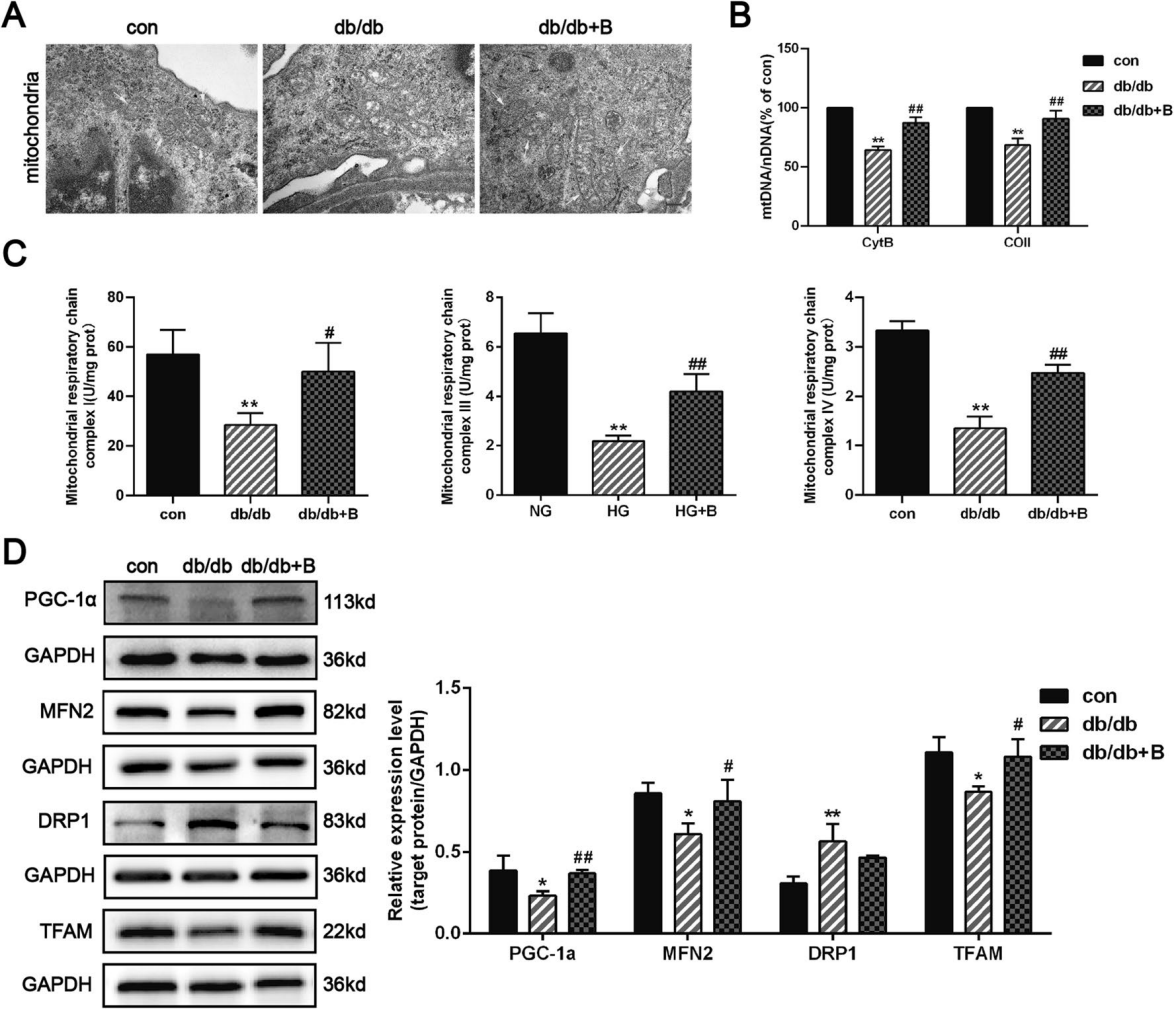
Effect of BSTL on mitochondrial biogenesis and dysfunction in vivo. **A** TEM of mitochondria from different groups (× 50 000). Scale bar, 1 µm. Arrow means representative pathological changes. **B** Quantitation of mtDNA content in the kidneys of mice from different groups. **C** Quantitation of mitochondrial respiratory chain complexes I, III, and IV activities in the kidneys of mice. **D** Representative western blots and quantitative assessment of PGC-1α, MFN2, DRP1, and TFAM in the kidneys of mice. ^*^P < 0.05 vs. con; ^**^P < 0.01 vs. con; ^#^*P* < 0.05 vs. db/db; ^##^*P* < 0.01 vs. db/db. con, the control mice; db/db, db/db mice; db/db + B, db/db mice treated with BSTL

As shown in Fig. 5A, TEM revealed mitochondrial damage in the podocytes of db/db mice, as shown by changes in mitochondrial shape, size, and organization of cristae. To determine the mitochondrial biogenesis capacity, we measured the mtDNA content and found a decreased mtDNA to nDNA ratio in the renal cortex tissues of db/db mice compared with the control mice. Subsequently, the activities of mitochondrial respiratory chain complexes I, III, and IV, which include proteins encoded by mtDNA, were significantly decreased in the kidneys of db/db mice. Effectively, BSTL treatment attenuated the decreased relative mtDNA content and rescued the activities of mitochondrial respiratory chain complexes I, III, and IV (Fig. 5B, C). At the molecular level, the protein expression levels of the mitochondrial biogenesis key protein PGC1-α, mtDNA replication/translation key protein TFAM, and mitochondrial fusion protein MFN2 were significantly decreased, while those of the mitochondrial fission protein DRP1 were increased in db/db mice compared with the control mice. BSTL significantly restored the protein expression levels of PGC1-α, TFAM, and MFN2; as for DRP1, BSTL reduced its expression level, however, with no statistically significant difference (Fig. 5D).

**Fig. 6:**
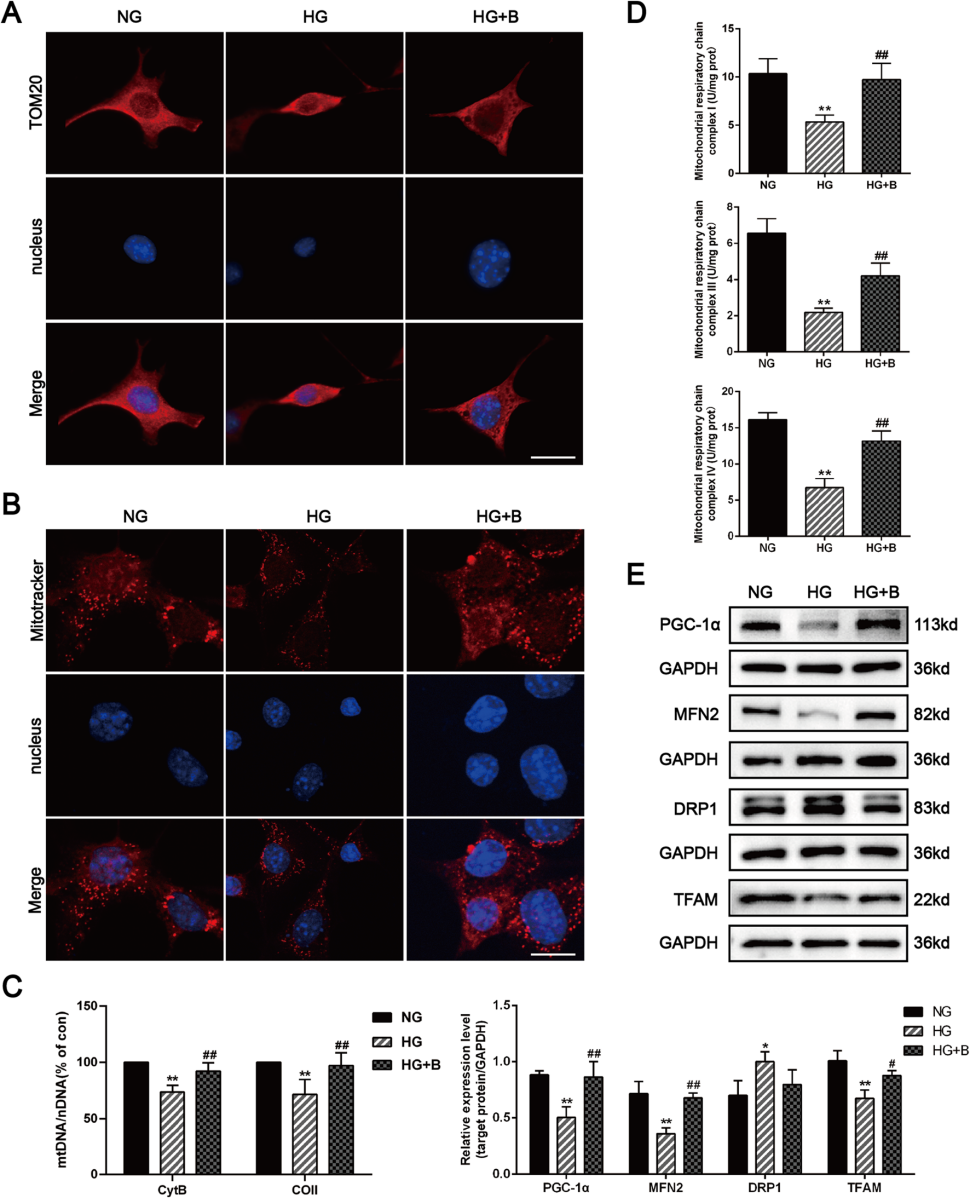

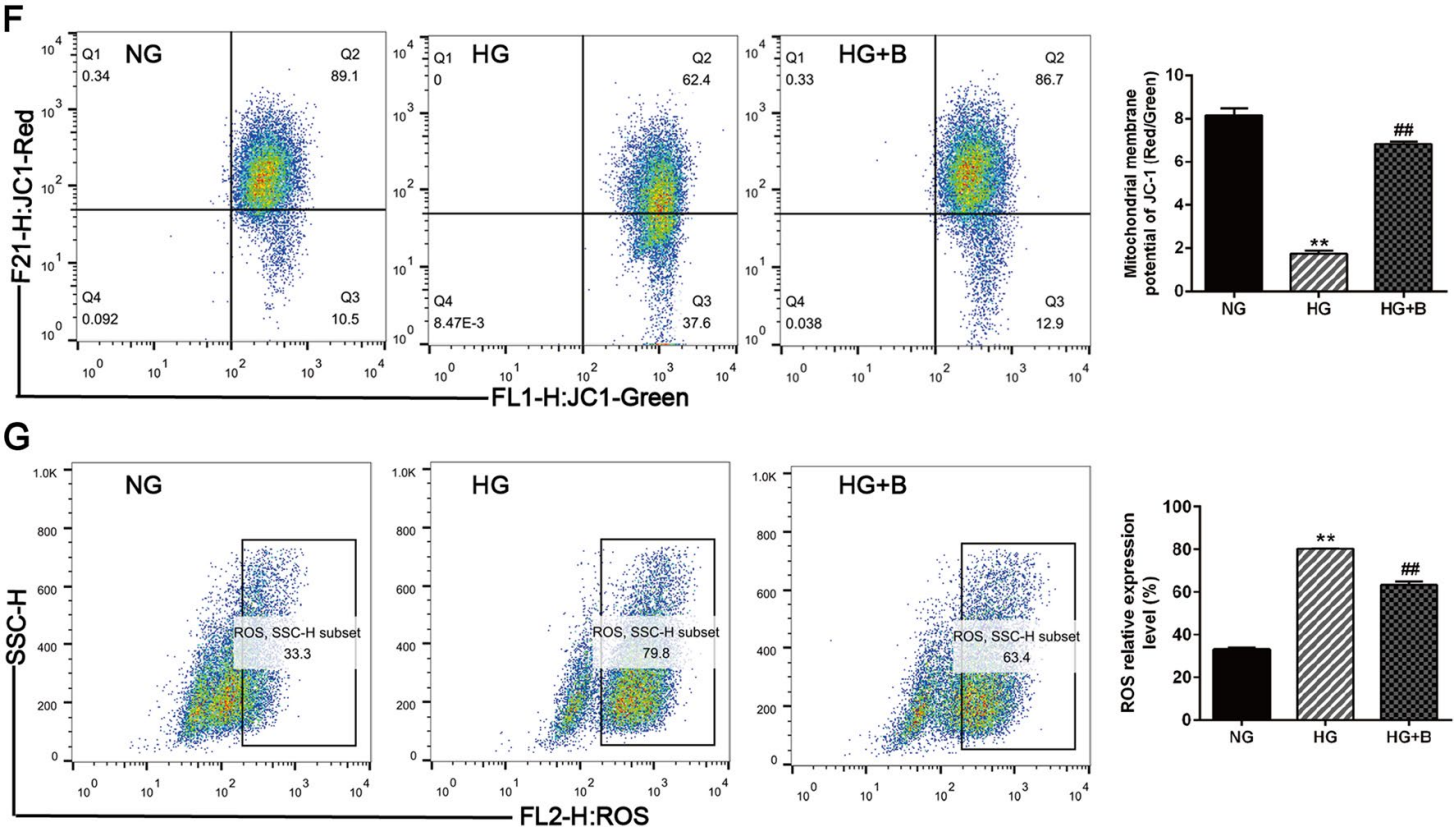
Effect of BSTL on mitochondrial biogenesis and dysfunction in vitro. **A** Immunofluorescence results of TOM20 in podocytes from different groups (× 400). Scale bar, 30 µm. **B** Immunofluorescence results of MitoTracker in podocytes from different groups (× 400). Scale bar, 30 µm. **C** Quantitation of mtDNA content in podocytes from different groups. **D** Quantitation of mitochondrial respiratory chain complexes I, III, and IV activities in podocytes from different groups. **E** Representative western blots and quantitative assessment of PGC-1α, MFN2, DRP1, and TFAM in podocytes from different groups. **F** Representative flow cytometry analysis depicting the detection of MMP in podocytes with different treatments, and quantitative data expressing the overall percentage of cells apoptotic and necrotic (Q2 represented the ratio of red fluorescence, Q3 represented the ratio of green fluorescence, Q2/Q3 represented the MMP). **G** Representative flow cytometry scatter plots and quantitative assessment of ROS production in podocytes from different groups. ^*^P < 0.05 vs. NG; ^**^P < 0.01 vs. NG; ^#^*P* < 0.05 vs. HG; ^##^*P* < 0.01 vs. HG. NG, normal glucose; HG, high glucose; HG + B, high glucose combined with BSTL drug-containing serum

TOM20 and MitoTracker staining were used to label mitochondria, and this staining revealed that podocytes had a decreased number of mitochondria under HG conditions; BSTL treatment reversed the HG-induced mitochondrial abnormalities by maintaining mitochondrial quantity in podocytes (Fig. 6A, B). Our in vitro study further confirmed the effect of BSTL in improving mitochondrial biogenesis and podocyte dysfunction. Consistent with the findings of animal experiments, we found that HG significantly reduced mtDNA copy numbers, mitochondrial respiratory chain complexes I, III, and IV activities and PGC1-α, TFAM, and MFN-2 protein levels and increased DRP1 levels in podocytes; all of these effects, except for the increased DRP1 levels, were markedly reversed by BSTL treatment (Fig. 6C–E). Moreover, we found that the MMP was significantly decreased and ROS production was markedly increased in HG-treated podocytes compared with the control cells; however, BSTL restored the MMP and inhibited the excessive ROS production in HG-treated podocytes. These results suggest that BSTL effectively protects the mitochondria by maintaining mitochondrial biogenesis in DKD (Fig. 6F, G).

### BSTL restored mitochondrial biogenesis in podocytes by regulating the AMPK pathway

**Fig. 7:**
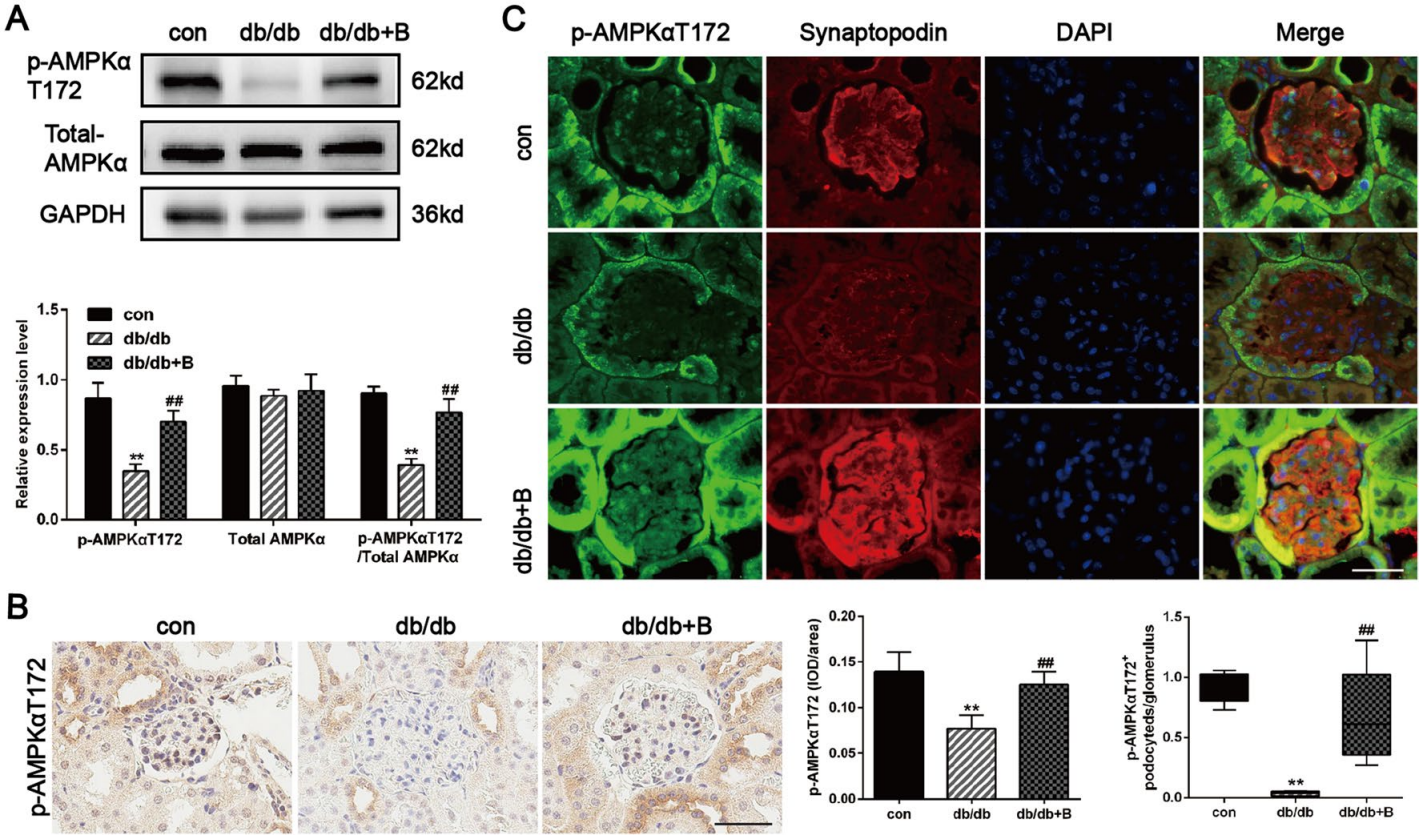
Effect of BSTL on the expression of AMPK in vivo. **A** Representative western blots of p-AMPKαT172 and total AMPKα in the kidneys of mice and quantitation of these results. **B** IHC staining of renal sections for p-AMPKαT172 in different groups, then assessed quantitatively for them (× 400). Scale bar, 30 µm. **C** Representative images of double immunofluorescent staining of glomerular synaptopodin and p-AMPKαT172 in different groups and quantitation of these results (× 400). Scale bar, 30 µm. ^*^P < 0.05 vs. con; ^**^P < 0.01 vs. con; ^##^*P* < 0.01 vs. db/db. con, control mice; db/db, db/db mice; db/db + B, db/db mice treated with BSTL

To explore the molecular mechanisms by which BSTL induced mitochondrial biogenesis in podocytes, we next assessed the status of AMPKα under diabetic conditions. Our results revealed that the phosphorylation of AMPKα at T172 was inhibited in the kidneys of db/db mice (Fig. 7A). Consistently, the diabetic mice displayed a reduced level of p-AMPKαT172 in the glomeruli compared with the control mice, and this effect was significantly reversed by BSTL treatment (Fig. 7B). To further measure p-AMPKαT172 expression in diabetic podocytes, we detected the expression of synaptopodin and p-AMPKαT172 by using double immunofluorescence staining. As shown in Fig. 7C, db/db mice exhibited less colocalization of p-AMPKαT172 and synaptopodin in the glomeruli than the control mice, indicating that db/db mice had decreased expression of p-AMPKαT172 in podocytes. After BSTL treatment, p-AMPKαT172 expression in podocytes was significantly restored.

**Fig. 8:**
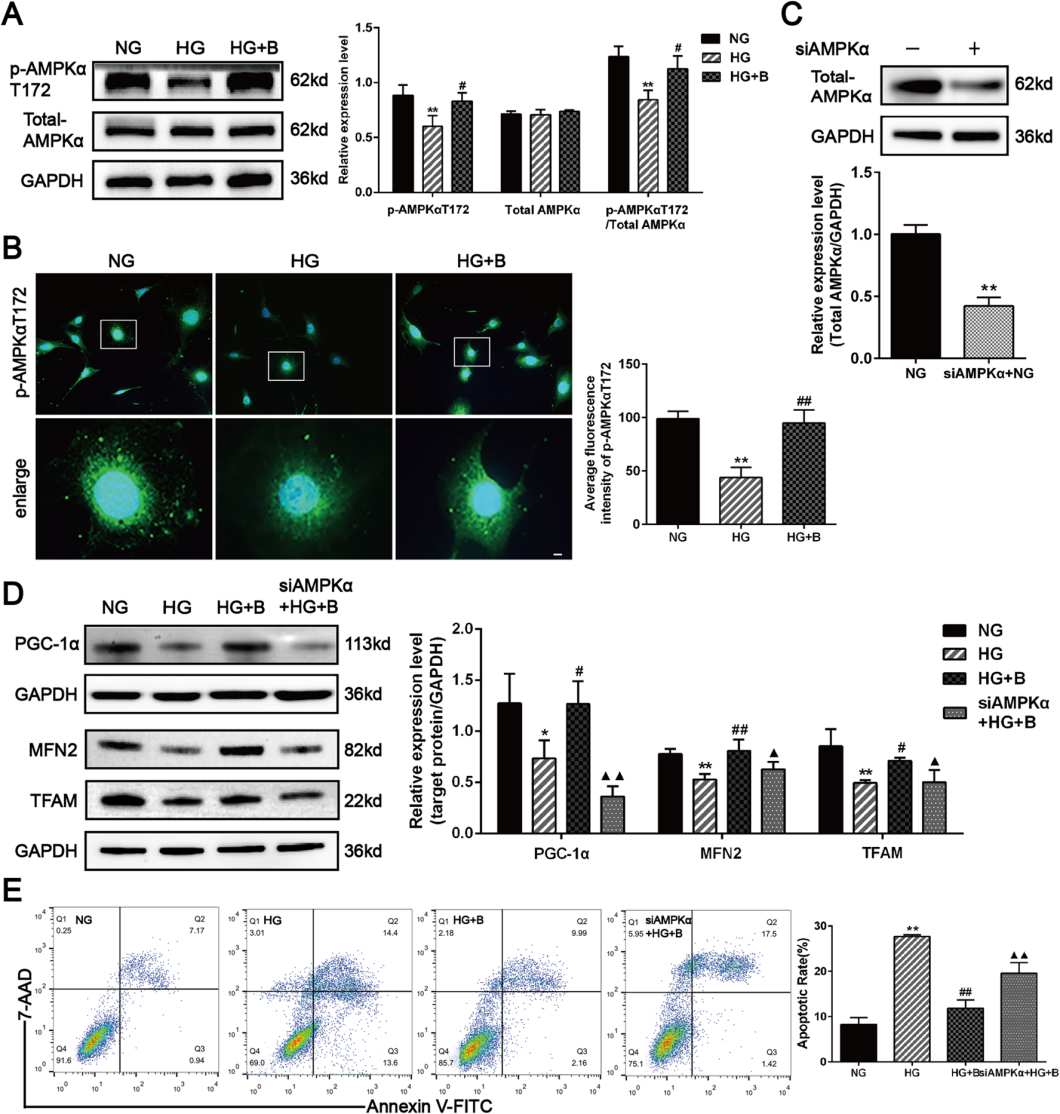
Effects of AMPKα siRNA on mitochondrial biogenesis and podocyte apoptosis in BSTL treated podocytes under HG conditions. **A** Representative western blots of p-AMPKαT172 and total AMPKα in podocytes and quantitation of these results. **B** Immunofluorescence staining of podocytes for p-AMPKαT172 in different groups, then assessed quantitatively for them (× 400, × 2000). Scale bar, 30 µm. **C** Representative western blots images and quantitation of siAMPKα expression in podocytes after the indicated treatments. **D** Representative western blots and quantitative assessment of PGC-1α, MFN2, and TFAM in podocytes after silenced AMPKα. **E** Representative flow cytometry analysis depicting the detection of apoptosis in podocytes with different treatments, and quantitative data expressing the overall percentage of podocyte apoptosis (Q2 represented the ratio of late apoptotic cells, Q3 represented the ratio of early apoptotic cells, Q2 + Q3 represented the total ratio of the apoptotic cells). ^*^P < 0.05 vs. NG; ^**^P < 0.01 vs. NG; ^#^*P* < 0.05 vs. HG; ^##^*P* < 0.01 vs. HG; ^▲^*P* < 0.05 vs. HG + B; ^▲▲^*P* < 0.01 vs. HG + B. NG, normal glucose; HG, high glucose; HG + B, high glucose combined with BSTL drug-containing serum. siAMPKα + B, silenced AMPKα in HG condition and treated with BSTL drug-containing serum

Finally, we determined the role of BSTL-regulated AMPK signalling pathway in promoting mitochondrial biogenesis and ameliorating podocyte toxicity under HG conditions. Our findings showed that the protein level of p-AMPKαT172 was significantly decreased in HG-treated podocytes, while BSTL significantly increased p-AMPKαT172 expression, which was consistent with the immunofluorescence results (Fig. 8A, B). Then, we silenced AMPKα in podocytes by transfection with a specific siRNA that targeted AMPKα, and endogenous AMPKα expression was markedly decreased, as shown in Fig. 8C. The renoprotective effect of BSTL on the protein expression levels of PGC-1α, MFN2, and TFAM was partially abolished in AMPKα siRNA-treated podocytes (Fig. 8D). Additionally, inhibition of AMPKα expression impaired the protective role of BSTL in HG-induced podocyte toxicity, as shown by the percentage of apoptotic cells. The results suggest that the BSTL-induced mitochondrial biogenesis in podocytes under HG conditions depends on the AMPK pathway, and this mechanism alleviates DKD podocyte injury (Fig. 8E).

## Discussion

BSTL is a compound formula which contains seven medical botanical drugs, according to the TCM theory, it has the effect of tonifying the kidney, activating blood, and dredging collaterals. Previous studies have identified its effect on DKD. Through the HPLC–ESI–MS analysis, we obtained the main active substances of the BSTL, including cryptotanshinone, rosmarinic acid, salvianolic acid A, tanshinone IIA, apigenin and astragaloside IV, etc. Among all 48 components, more than half of them have regulatory effects on mitochondria. For example, studies have shown that tanshinone IIA [27] and apigenin [23] can protect the mitochondria by inhibiting mitochondrial oxidative stress and improving mitochondrial dysfunction in many diseases. Cryptotanshinone [24], rosmarinic acid [25] and salvianolic acid A [26] also has been reported to be useful in promoting mitochondrial biogenesis by activating AMPK pathway. Moreover, astragaloside IV could protect podocytes from injury via ameliorating mitochondrial dysfunction in diabetic rats, as demonstrated in previous studies [28]. Therefore, we assume that these active compounds may be involved in the podocyte protection effect of BSTL by improving mitochondrial dysfunction in DKD. Podocytes that are exposed to hyperglycaemia often undergo a variety of pathological changes, including hypertrophy, dedifferentiation, foot process effacement, detachment, loss, and apoptosis, thus resulting in proteinuria and glomerulosclerosis, which are representative features of DKD [29–32]. Of note, podocyte damage often occurs in the early stage of DKD [33]. In our study, db/db diabetic mice developed overt proteinuria with major glomerular lesions, but the mice did not exhibit significant changes in the Scr levels; these results are consistent with the early manifestations of DKD. From the results, it is clear that BSTL significantly reduced the UACR and attenuated the renal histological lesions in db/db mice. Consistently, we demonstrated that BSTL prevented podocyte damage in both db/db diabetic mice and HG-treated podocytes. BSTL significantly improved podocyte foot process fusion and effacement in db/db mice and inhibited actin cytoskeleton rearrangement in HG-induced podocytes. Moreover, BSTL restored the protein expression of nephrin and podocin, which are key proteins of the glomerular filtration barrier, and decreased podocyte apoptosis in both in vivo and in vitro studies.

Podocytes are rich in mitochondria, which are double-membraned organelles with abundant cristae that provide sites for cellular respiration and adenosine triphosphate (ATP) production via oxidative phosphorylation (OXPHOS) [34]. Under diabetic or hyperglycaemic states, excessive glucose enters the tricarboxylic acid cycle, resulting in a greater number of protons entering the mitochondrial electron transport chain [35]; however, damaged respiratory chain complexes cause abnormally high proton leakage across the inner mitochondrial membrane to produce excessive ROS [36], which are primarily produced by redox-active components that are involved in the mitochondrial respiratory chain [37, 38]. Moreover, increased uncoupling of the respiratory chain resulting from a decreased MMP also leads to diminished ATP synthesis by mitochondria under diabetic conditions [39]. Previous studies described that mice with diabetes mellitus and podocytes treated with HG exhibited aberrant mitochondrial structure and function, such as fragmented morphology, decreased number of mitochondria, decreased MMP and excessive ROS production, which further damaged the podocytes [40]. Similarly, our study revealed mitochondrial abnormalities, including a decrease in mitochondrial number, abnormalities in mitochondrial morphology, and impaired activity of mitochondrial respiratory chain complexes I, III, and IV, in db/db mice and HG-treated podocytes. We also observed decreased MMP, which is another marker of apoptosis, and excessive ROS production in HG-treated podocytes. However, BSTL effectively improved these mitochondrial abnormalities, increased the activities of mitochondrial respiratory chain complexes I, III, and IV, restored the MMP, and inhibited excessive ROS production. These findings indicated that BSTL prevents the progression of DKD by positively regulating the mitochondrial function of podocytes.

Increasing numbers of publications have reported that impaired mitochondrial functions are involved in various diseases, such as diabetes, Parkinson's disease, and nonalcoholic fatty liver disease [41–44]. Mitochondrial biogenesis is the process by which new mitochondria are formed in tissues and cells, and this process is activated by different signals in response to internal and external environmental stimuli [45]. PGC-1α, which is an upstream transcriptional regulator of mitochondrial biogenesis [41], regulates TFAM to promote mitochondrial biogenesis, thus increasing mtDNA replication and transcription and ultimately leading to an increased number of mitochondria and enhanced OXPHOS function [46]. TFAM is also a major regulator of mtDNA copy number, and TFAM deficiency has been linked to lower production of mtDNA-encoded proteins and lower OXPHOS capability [47]. Studies have shown that chronic hyperglycaemia reduces the expression levels of PGC-1α and TFAM [48], while activation of PGC1-α and increased expression of TFAM protect against HG-mediated podocyte injury by promoting mitochondrial biogenesis [49]. Additionally, the normal process of mitochondrial biogenesis is inseparable from the dynamic balance of mitochondrial fusion and fission [50]. Mitochondria meet the metabolic and energy needs of cells by constantly changing and remodelling their shape [51]. When tissues and cells are stimulated by internal and external factors, DRP1 is transported from the cytoplasm to the outer mitochondrial membrane and assembled into a circular polyplex structure. Then, a guanosine triphosphatase (GTP)-dependent mechanism activates the downstream mitochondrial fission factors to drive mitochondrial breakage [52], and MFN2 is embedded in the outer mitochondrial membrane to regulate mitochondrial fusion [53]. Increasing evidence suggests that mitochondrial biosynthesis is reduced in HG environments [54], and HG increases the expression of mitochondrial fission proteins, while inhibits the expression of mitochondrial fusion proteins in renal tissues [55]. Our previous study found that mitochondrial autophagy impairment in podocytes under HG conditions, and BSTL treatment promotes mitochondrial autophagy to alleviate podocyte injury. In this study, we found that the decrease in the mtDNA content was accompanied by a reduction in the protein expression of PGC-1α and TFAM in db/db mice and HG-treated podocytes, while BSTL increased the mtDNA copy numbers and the expression of mitochondrial biogenesis-related proteins. Our study also showed increased DRP1 protein expression and decreased MFN2 protein expression in db/db mice and HG-treated podocytes. However, BSTL promoted MFN2 protein expression but had no significant effect on DRP1 levels. These results suggest that BSTL attenuated podocyte mitochondrial dysfunction by activating mitochondrial biogenesis.

Mechanistically, AMPK is a key regulator in the maintenance of cellular metabolism, and it is involved in cellular activities such as cell proliferation, cell cycle progression, and apoptosis [56]. Increasing numbers of studies have suggested that some agents, such as pyrroloquinoline, quinine, and nanomitochondria N-tert-butyl-α-phenylnitrone (MitoPBN), as well as natural products, such as catalpol and hydroxytyrosol, mitigate Parkinson's disease and diabetes by improving mitochondrial biogenesis via the AMPK signalling pathway [42, 57–59]. AMPK increases mitochondrial biogenesis by directly regulating PGC-1α expression, thus improving the OXPHOS capacity of tissues and cells [60]. Moreover, AMPK augments glucose metabolism, inhibits oxidative stress, and elevates MMP by regulating MFN2 to improve mitochondrial fusion and mitochondrial biogenesis function during mitochondrial energy crises [61]. Previous studies showed decreased p-AMPK levels in HG-treated podocytes [62]. Our study indicated that BSTL improved p-AMPKα expression in vitro and in vivo. In contrast, the renoprotective effects of BSTL on the protein expression of PGC-1α, MFN-2, and TFAM as well as podocyte apoptosis were partially abolished in AMPKα siRNA-treated podocytes. These results indicate that BSTL may protect against DKD podocyte injury by promoting mitochondrial biogenesis through the regulation of the AMPK signalling pathway.

However, our study has some limitations. Firstly, we did not perform AMPKα knockdown experiments in vivo. Secondly, mitochondrial oxygen consumption rates and ATP contents were not measured in our study. Thirdly, our results confirm the effect of BSTL on AMPK, but not enough to determine whether it is a direct or indirect target. In addition, the extraction and isolation of monomers of TCM compounds that protect mitochondria may provide a further base for the prevention and treatment of podocyte injury in DKD.

## Conclusions

We revealed that BSTL alleviated podocyte injury and repaired mitochondrial damage in db/db mice and HG-treated podocytes and that promoting mitochondrial biogenesis through AMPK regulation was one of the important potential underlying mechanisms. On the one hand, BSTL directly increased mitochondrial biosynthesis by activating AMPK; on the other hand, BSTL promoted mitochondrial biogenesis by regulating the mitochondrial fusion process. In summary, our study provides a feasible clinical application for the prevention and treatment of DKD and identifies a potential mechanism underlying this effective therapy. Our study has important significance for future research and treatment of DKD.

## Data Availability

The data used to support the findings of this study are available from the corresponding author upon request.

## Abbreviations

Akt: Protein kinase B
ALT: Alanine aminotransferase
AMPK: Adenosine monophosphate-activated protein kinase
AMPKα siRNA: AMPKα small interfering RNA
ANOVA: One-way analysis of variance
AST: Aspartate aminotransferase
BCA: Bicinchoninic acid
BSTL: BaoShenTongLuo
BUN: Blood urea nitrogen
COII: Cytochrome c oxidase subunit II
Cyt B: Cytochrome b
DAB: Diaminobenzidine
DAPI: 4’,6-Diamidino-2-phenylindole
DKD: Diabetic kidney disease
DRP1: Dynamin-related protein 1
ECL: Enhanced chemiluminescence
Epon: Epoxy resin
ESI: Electrospray ionization
ESRD: End-stage renal disease
FITC: Fluorescein isothiocyanate
HE: Haematoxylin and eosin
HG: High glucose
HPLC–ESI–MS: High-Performance Liquid Chromatography Electrospray Ionization Mass Spectrometer
HRP: Horseradish peroxidase
IBM SPSS: International Business Machines Corporation Statistical Product and Service solutions
IFN: Interferon
IHC: Immunohistochemical
IQR: Interquartile range
LSD: Least significant difference
MFN2: Mitochondrial fusion protein 2
MMP: Mitochondrial membrane potential
MPC5: Mouse podocyte clone-5
mtDNA: Mitochondrial deoxyribonucleic acid
nDNA: Nuclear DNA
NG: Normal glucose
PAS: Periodic acid-Schiff
PBS: Phosphate-buffered saline
PBST: Phosphate-buffered solution
PGC-1α: Peroxisome proliferator-activated receptor gamma coactivator 1 alpha
PI: Propidium iodide
PI3K: Phosphoinositide 3-kinase
qPCR: Quantitative real-time PCR
ROS: Reactive oxygen species
RPMI: Roswell Park Memorial Institute
rTdT: R-terminal deoxynucleotidyl transferase
Scr: Serum creatinine
SD: Sprague‒Dawley
SIR: Selected ion recording
SSC: Saline sodium citrate
TCMs: Traditional Chinese medicines
TEM: Transmission Electron Microscopy
TFAM: Transcription factor A
TOM20: Translocase of outer mitochondrial membrane 20 homologue
TUNEL: Terminal Deoxynucleotidyl Transferase-Mediated dUTP-biotin Nick end Labelling
UACR: Urinary albumin/creatinine ratio
WB: Western Blotting
